# Supplementary material for: Dynamic Regulation of miRNA Expression by Functionally Enhanced Placental Mesenchymal Stem Cells PromotesHepatic Regeneration in a Rat Model with Bile Duct Ligation
Source: Int J Mol Sci. 2019 Oct 24;20(21):5299. doi: 10.3390/ijms20215299 (PMC6862171; doi:10.3390/ijms20215299)
Supplement: Supplementary file 1 [file ijms-20-05299-s001.pdf]

Supplementary

Table S1. Primer sequences for human using quantitative real time polymerase chain reaction.

Gene	Accession Number	Sequence	Tm (℃)
HIF1A	NM_001530.4	F: 5'-GTTTACTAAAGGACAAGTCA-3'<br>R: 5'-TTCTGTTTGTTGAAGGGAG-3'	60
VEGF	NM_001204384.1	F: 5'-GCCTTGCCTTGCTGCTCTAC-3'<br>R: 5'-ACATCCATGAACTTCACCACTTCG-3'	60
PRL-1	NM_003463.4	F: 5'-TACTGCTCCACCAAGAAGCC-3'<br>R: 5'-AGGTTTACCCCATCCAGGTC-3'	60
ITGA4	NM_000885.6	F: 5'-TTCCTACGGGCTGTGTTC-3'<br>R: 5'-CTGAAGTTTGCCAGTTTGG-3'	55
ITGB7	NM_000889.3	F: 5'-AGCAGCAACAACTCAACTGG-3'<br>R: 5'-TTACAGACCCACCCTTCCTCT-3'	55
RHOA	NM_001664.4	F: 5'-TGGAAAGCAGGTAGAGTTGG-3'<br>R: 5'-GACTTCTGGGGTCCACTTTT-3'	55
ROCK1	NM_005406.3	F: 5'-GAAGAAAGAGAAGCTCGAGA-3'<br>R: 5'-GATCTTGTAGCTCCCGCATCTGT-3'	55
GAPDH	NM_002046.7	F: 5'-GCACCGTCAAGGCTGAGAAC-3'R: 5'-<br>GTGGTGAAGACGCCAGTGGA-3'	60

Table S2. Primer sequences for rat using quantitative real time polymerase chain reaction.

Gene	Accession Number	Sequence	Tm (℃)
ITGA4	NM_001107737.1	F: 5'-GGAAGCCCCAGTGGAGAAC-3'<br>R: 5'-ATTGTCACTCCCAGCCACTGA-3'	55
ITGA6	NM_053725.1	F: 5'-AGCCCCAGGGACTTACAACT-3'<br>R: 5'-CTTCATAGGGCCCATCTTCA-3'	55
ITGB1	NM_017022.2	F: 5'-AACAGTGAAGACATGGATGC-3'<br>R: 5'-CTCTCTCTTCCTGCACACAC-3'	55
ITGB7	NM_013171.1	F: 5'-AGTGCCCTCCAAGCTTAACCAC-3'<br>R: 5'-CGTCCCACCTCTCTCTCGAA-3'	55
ENG	NM_001010968.2	F: 5'-AAGGTGTGACTGGACACAAG-3'<br>R: 5'-CCAGATCTGCATATTGTGGT-3'	60
PDGFRA	NM_012802.1	F: 5'-GAGGACGATTCTGCCATCAT-3'<br>R: 5'-CAGTTCTGACGTGGCTTTCA-3'	60
PDGFRB	NM_031525.1	F: 5'-TGTTCGTGCTATTGCTCCTG-3'<br>R: 5'-TGTCAGCACACTGGAGAAGG-3'	60
IL-6R	NM_017020.3	F: 5'-CCTTGTAAATGCCTTTTGTG-3'<br>R: 5'-GTACACTTTGTCACCCTCCA-3'	60
HNF1A	NM_012669.1	F: 5'-AAGATGACACGGATGACGATGG-3'<br>R: 5'-GGTTGAGACCCGTAGTGTCC-3'	60
HNF4A	NM_022180.2	F: 5'-AAATGTGCAGGTGTTGACCA-3'<br>R: 5'-CACGCTCCTCCTGAAGAATC-3'	60
GAPDH	NM_017008.4	F: 5'-TCCCTCAAGATTGTCAGCAA-3'<br>R: 5'-AGATCCACAACGGATACATT-3'	60

Table S3. miRNA sequence and target gene in 3'-UTR site.

miRNA Target Gene	Accession Number	Target Sequence
hsa-miR-30a-5p	MIMAT0000087	3'-GAAGGUCAGCUCCU**ACAAAUGU**-5'
hPRL-1	NM_003463.4	5'-GCCUGCUCACUUUA**UGUUUACA**-3'
hITGA4	NM_000885.6	5'-AAUUUAAAAGACAC**UGUUUACA**-3'
hsa-miR-340-5p	MIMAT0004692	3'-UUAGUCAGAGUAACG**AAAUAUU**-5'
hITGA4	NM_000885.6	5'-CUUUAAAGCCCUUUA**UUUAUAA**-3'
hsa-miR-146a-3p	MIMAT0004608	3'-GACUUCUUGACUUA**AAGUCUC**C-5'
hITGB7	NM_000889.3	5'-CACCCUACUUCAUU**UUCAGAG**U-3'
rno-miR-340-5p	MIMAT0004650	3'-UUAGUCAGAGUAAC**GAAAUAUU**-5'
rITGB1	NM_017022.2	5'-UCCCAACGCCUUCU**CUUUAUAA**-3'
rno-miR-30a-5p	MIMAT0000808	3'-GAAGGUCAGCUCCU**ACAAAUGU**-5'
rITGA6	NM_053725.1	5'-UACAAAUGAUGCCU**UGUUUACA**-3'
rno-miR-27a-3p	MIMAT0000799	3'-CGCCUUGAAUCGGU**GACACUU**-5'
rPDGFRA	NM_012802.1	5'-GUCUUGGGAAGCCU**CUGUGAA**-3'
rno-miR-21-5p	MIMAT0000790	3'-AGUUGUAGUCAGAC**UAUUCGAU**-5'
rIL-6R	NM_017020.3	5'-CCUUUUGACUUUUU**AUAAGCUA**-3'
rno-miR-122-5p	MIMAT0000827	3'-GUUUGUGGUAACAGU**GUGAGG**U-5'
rHNF1A	NM_001530.4	5'-CCUGUGCCUCCCAGG**CCACUCC**A-3'
